# Superimposition of Viral Protein Structures: A Means to Decipher the Phylogenies of Viruses

**DOI:** 10.3390/v12101146

**Published:** 2020-10-09

**Authors:** Janne J. Ravantti, Ane Martinez-Castillo, Nicola G.A. Abrescia

**Affiliations:** 1 Molecular and Integrative Biosciences Research Programme, Faculty of Biological and Environmental Sciences, University of Helsinki, FI-00014 Helsinki, Finland; Janne.Ravantti@helsinki.fi; 2 Center for Cooperative Research in Biosciences (CIC bioGUNE), Basque Research and Technology Alliance (BRTA), Bizkaia Technology Park, 48160 Derio, Spain; amcastillo@cicbiogune.es; 3 IKERBASQUE, Basque Foundation for Science, 48013 Bilbao, Spain; 4 Centro de Investigación Biomédica en Red de Enfermedades Hepáticas y Digestivas (CIBERehd), Instituto de Salud Carlos III, 28029 Madrid, Spain

**Keywords:** virus evolution, structural similarity, double β-barrel fold, structure-based phylogeny

## Abstract

Superimposition of protein structures is key in unravelling structural homology across proteins whose sequence similarity is lost. Structural comparison provides insights into protein function and evolution. Here, we review some of the original findings and thoughts that have led to the current established structure-based phylogeny of viruses: starting from the original observation that the major capsid proteins of plant and animal viruses possess similar folds, to the idea that each virus has an innate “self”. This latter idea fueled the conceptualization of the PRD1-adenovirus lineage whose members possess a major capsid protein (innate “self”) with a double jelly roll fold. Based on this approach, long-range viral evolutionary relationships can be detected allowing the virosphere to be classified in four structure-based lineages. However, this process is not without its challenges or limitations. As an example of these hurdles, we finally touch on the difficulty of establishing structural “self” traits for enveloped viruses showcasing the coronaviruses but also the power of structure-based analysis in the understanding of emerging viruses

## 1. Introduction

Viruses mutate at a faster rate than cellular organisms—about four orders of magnitude [1]. Therefore the traceability of evolutionary relationships by sequence similarity is easily lost even within the same virus family. Sequence similarity can be assessed at DNA/RNA or protein level, and several tools have been developed to decipher whether two (or more) proteins are homologous or descend from a common ancestor (homology; divergent evolution) [2]. Interpretation of high sequence similarity between two proteins nevertheless cannot rule out the different scenario driven by convergent evolution (analogy or coincidence). Thus, common ancestry should never be inferred by using a single number or score, but rather, it should involve the fulfilment of other constraints such as the biological function, cellular localization, and/or the gene locus within the genome. In the virus world, some of these constraints become muddled-up and comparison of sequences (linear strings of a combination of either four (nucleotides) or twenty (amino acids) letters) becomes of limited usefulness for unravelling primitive precursors. On the other hand, the process of comparing three-dimensional (3D) objects is an activity that humans show at very young age, from about 3 years old, and which helps children (us, once upon a time) in establishing new object categories, an invaluable learning process of the outside world [3]. This same activity but comparing the structures of two or more proteins allows us to identify the salience of conserved 3D features even when the similarity in their primary sequences has become undetectable over the course of time. As the 3D structure entails structure-function constraints tuned along the course of evolution, the structure-based methods represent a powerful means for phylogenetic classification, not only of the protein world but also of the virosphere [4,5]. The elucidation of virus common ancestry together with current genome sequencing capabilities and the advance of cryo-electron microscopy (cryo-EM) for biomolecular structure determination [6,7] will also unlock the complexity of the intertwined evolutionary relationship with cellular organisms.

Here, we briefly review some of the groundwork concepts that have led to the quantitative comparison of 3D protein structures and its implications for the structure-based taxonomy of viruses as emerged from the initial systematic study of major capsid proteins (MCPs) possessing a double β-barrel topology (or double jelly-roll; DJR). Finally, we glance at the challenge of a structure-based classification for enveloped viruses and highlight the impact that structural comparisons entail on our understanding of how evolution can change structural motifs leading to the emergence of new viruses as in the case of SARS-CoV-2.

## 2. Structural Superimposition of Proteins and Homology of Viral Coat Proteins

The necessity of comparing 3D protein structures stretches back to the early sixties thanks to success in the determination of the hemoglobin crystal structure and the full establishment of macromolecular X-ray crystallography as a leading technique in the structural biology field [8,9]. In light of these results Huber and colleagues performed a structural alignment study between insect (invertebrate) hemoglobin and whale (vertebrate) myoglobin showing their high structural similarity despite the low amino acid sequence identity (~20%) [10]. They used a least-square minimization algorithm to minimize the sum of the square of the distances across the equivalent Cαs in the corresponding backbones [10]. The concept of superimposing equivalent residues when these are not visually intuitive is not trivial and so is the definition of a metric for the “goodness” of the alignment. A method for defining equivalent residues when these are not visually obvious (as in the case of myoglobins) was proposed in 1976 by Rossmann and Argos and aimed at identifying structural homology across proteins (HOMOLOGY software) [11,12]. They proposed to use a “probabilistic” approach to define the subset of equivalent backbone atoms in comparing sets to overcome the difficulty of this assignment. Once these were defined, the degree of structural parallelism was calculated. Since then several tools such as COMPARER [13], Secondary Structure Matching (SSM) [14], Dali [15], Structure Homology Program (SHP) [16], and Homologous Structure Finder (HSF) [17], to mention a few, have been developed to address the structural superimposition problem. The “probabilistic” method proposed by Rossmann and Argos is at the base of some of these tools such as SHP and HSF software which have been extensively used for establishing the structure-based classification of viruses (see below).

**Figure 1:**
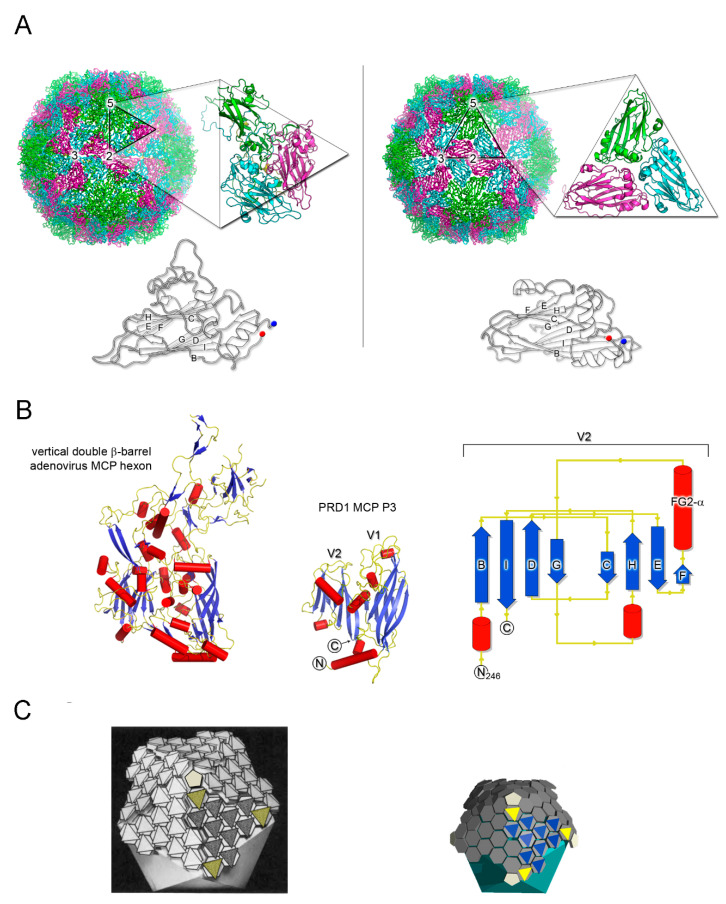
Detection of structural homology in MCPs. (**A**) Side-by-side comparison of capsid organization of human rhinovirus 14 (left; HRV-14, PDBid 1NA1) and southern beam mosaic virus (right; SBMV, PDBid 4SBV) represented to scale as cartoon tube. Insets, icosahedral asymmetric units (marked with a black triangle) of HRV-14 composed of MCPs VP1 (green), VP2 (magenta), VP3 (cyan), and VP4 (located beneath VP1-3 and only partially visible in yellow) and of SBMV composed of three copies of the coat protein, respectively, (depicted in cartoon). Below, comparison of HRV-14 VP2 and a copy of the SBMV coat protein as outlined cartoon with BIDG and CHEF sheets labelled; blue and red circles mark the N- and C-terminal ends, respectively. The numbers 5-, 3-, and 2- on the virions show the location of the 5-fold, 3-fold, and 2-fold icosahedral symmetry axes, respectively. (**B**) Vertical double β-barrel fold of MCPs of adenovirus hexon (left; PDBid 1P2Z) and of PRD1 P3 (center; PDBid 1CJD); both have been represented by secondary structural elements (red cylinders, α-helices; blue-arrows, β-strands; yellow, loops); right, topology diagram of the V2 single β-barrel (residues 246-383) of PRD1 P3 MCP. (**C**) Side-by-side comparisons, approximately to scale, of virion architectures of adenovirus (left; adapted from [22] with permission from Elsevier) and PRD1 (right). PRD1 representation has been inspired by the original geometric model of adenovirus (left) but derived from the coordinates of the PRD1 virion atomic model (PDBid 1W8X; [23,24]) with solid hexagons (grey) to represent the pseudo-hexameric geometry adopted by the capsomers which in turn are P3 trimers. Oligomerisation is depicted by flat triangles coloured in blue and yellow (peripentonal) for a virus facet; at the five-fold vertices pentagons (coloured in white smoke) to represent the penton proteins, as a solid cyan icosahedron the membrane vesicle beneath the capsid.

The eighties saw further successes of virus crystallography with the determination of plant viruses such as *Tomato bushy stunt virus* and *Southern bean mosaic virus* [18,19]. Later on animal-infecting viruses of the *Picornaviridae* family revealed noticeable structural similarities in the corresponding MCP folds despite the lack of sequence similarity—strikingly not only within viruses infecting the same host but also across viruses infecting different hosts [20,21]. These MCPs possessed eight β-strands arranged with the long axis of the β-barrel tangentially to the virus surface (Figure 1A). At the time, there were not many virus structures solved and therefore it was not clear whether the detected similarities were the remains of a common origin (divergent evolution) or they appeared simply because there was no other way of forming a suitable viral capsid (convergent evolution). All the above viruses were relatively small icosahedral particles (about 300 Å in diameter) possessing a single-stranded (ss) RNA genome. The similarity of the three MCPs, VP1, VP2, and VP3, of human rhinovirus 14 to the structure of those of the plant viruses led Rossmann and colleagues to put forward the idea that they diverged from a common ancestor (Figure 1A) [20]; at the time a hypothesis that—as we now realise—led to several important implications in virus phylogeny.

## 3. Major Capsid Proteins with a Double β-Barrel Fold: The Conceptualization of Viral Lineages

The observation suggested by Rossmann and co-workers that certain viruses may share a common descent pullulated the field for some time. In 2003, thanks to the boost of viral structures determined by X-ray, Chapman and Liljas carried out a comprehensive analysis of the topology of viral protein folds available [25]. Significantly, they noted the recurrence of certain folds in virions belonging to distinct taxonomic viral families and postulated the ramifications that this recurrence could have for inferring phylogenetic relationships [25]. Nonetheless, a systematic and unifying approach through which these ideas could be tested was still lacking, at least until the X-ray structure of the MCP P3 of lipid-containing phage PRD1 was determined [26]. The structure showed that both bacteria-infecting PRD1 and eukaryotic-infecting adenoviruses possess a similar MCP with a vertical double β-barrel fold, a finding that opened up a treasure chest of new discoveries. This fold entails two single β-barrel connected by a short linker; each β-barrel consists of eight β-strands arranged in two four-stranded antiparallel β-sheets packed together and with their long axis perpendicular to the capsid shell (Figure 1B). In opposition to all previously studied viruses with tangential β-barrels MCPs (e.g., Figure 1A), both PRD1 and adenovirus pointed at a different capsomer morphology and organization. The capsomers displayed a pseudo-hexameric appearance generated by the trimerization of the corresponding MCPs (Figure 1C); they could also be readily fitted into the low resolution cryo-EM map of *Paramecium bursaria Chlorella virus* (PBCV-1), a virus infecting algae [27].

**Figure 2:**
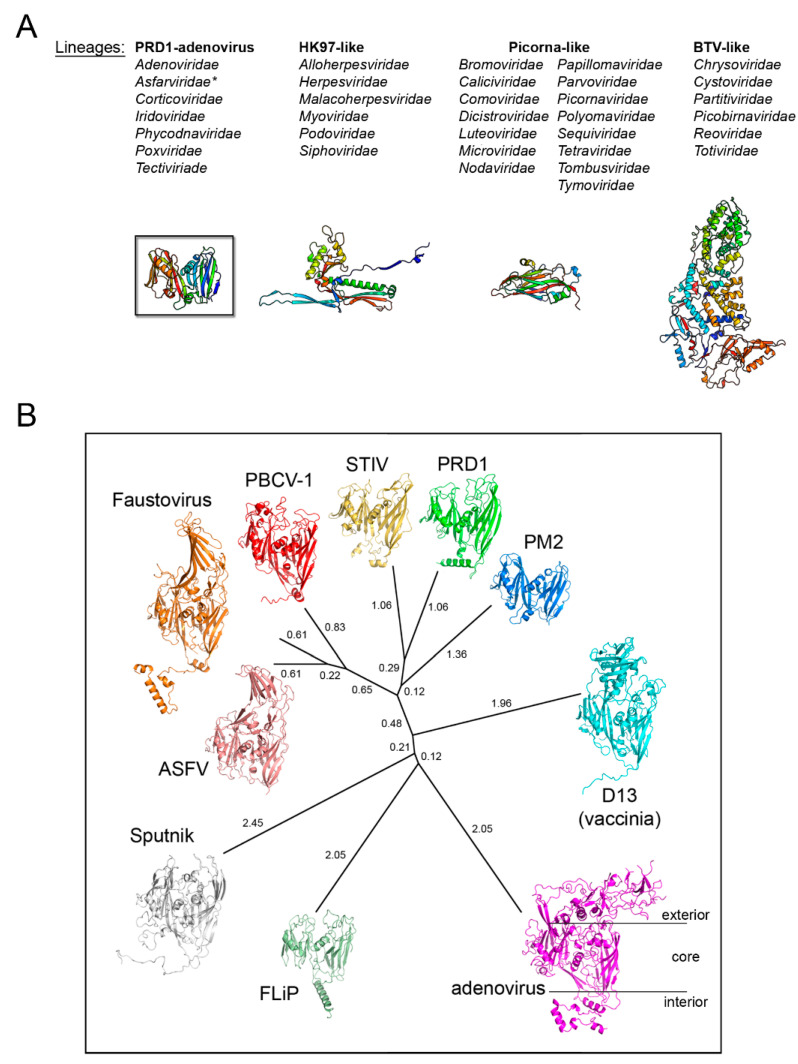
Structure-based viral phylogeny. (**A**) The four structure-based lineages representing 33 (+1) viral families with the *Asfarviridae* (*) recently added. Below, the fold of the MCP representative for each lineage depicted in cartoon and colored rainbow from blue to red from the N- to the C-terminal end. (**B**) Structure-based phylogenetic tree of the PRD1-adenovirus lineage. The evolutionary distances across all members (PRD1: PDBid 1HX6; PBCV-1: 1M3Y; adenovirus: 1P2Z; STIV: 2BBD; Vaccinia D13: 2YGB; Sputnik: 3J26; Faustovirus: 5J7O; FLiP: 5OAC; ASFV p72: 6KU9; PM2: 2W0C) were calculated with HSF [17] and depicted with PHYLIP software (https://evolution.genetics.washington.edu/phylip.html); the evolutionary distances are shown next to each branch. The black horizontal lines on the adenovirus hexon MCP highlights the loops elaboration on top of the jelly rolls, the core double β-barrel, and at the bottom extensions at the N- and C-terminal.

On the basis that coat protein topology and virion architecture could be used as fossil fingerprints (as “innate self” traits of these viruses), it was proposed that PRD1, adenovirus, and PBCV-1, the largest of the three with a diameter of ~1900 Å, would constitute a viral lineage [27]. Confirmation of this proposal came with the crystal structure of PBCV-1’s coat protein Vp54 as possessing a double β-barrel fold by the Rossmann group [28], and with the determination of the X-ray structure of the whole PRD1 at 4.2 Å resolution (Figure 1C, right) [23,24]. Several MCPs and virion structures elucidated to atomic details have expanded our understanding of the virosphere [28,29,30,31,32,33,34,35]. A clear example of this endeavor are the coat proteins with the so-called HK97 fold characterized basically by two domains: the P-domain, with a long α-helix and a three-stranded β-sheet, and the A-domain, with two α-helices and a β-sheet, and found in viruses infecting different domains of life [36]. With the accumulation of structural data, the idea emerged that viruses could be structurally classified in four lineages, initially called PRD1-like, HK97-like, BTV-like, and picorna-like [37]. The classification of the virosphere using the above four structure-based viral lineages accounts for more than 30 viral families (Figure 2A), in contrast to the 22 accounted by the five-rank Linnean-like structure used by the International Committee on Taxonomy of Virus (ICTV) until 2017 [38,39]. Very recently, however, in light of the accruing of structure-function and phylogenomic evidence of evolutionary relationships among what had been considered “distantly related viruses” (thanks also to the increased speed of genome sequencing, computing power, and more sensitive algorithms) has made unavoidable the revisiting of the virus classification [40,41,42,43]. Indeed, the ICTV has recognized the usefulness of extending its Code to a 15-rank classification hierarchy which reflects the proposal of a genome-based megataxonomy of the virus world that introduce the ranks of Realm, Kingdom, and Phylum among others [44,45].

Today, the first established structure-based PRD1-adenovirus lineage accounts for over 10 virus members infecting organisms across the three domains of life (Figure 2B). Remarkably, this lineage includes ssDNA viruses such as *Flavobacterium*-infecting, lipid-containing phage (FLiP) [46], non-icosahedral viruses such as Vaccinia [32,35], and virophages such as Sputnik, a satellite non-membrane-containing virus of the giant Mimivirus [47], which leads us to the question of whether or when—as the descent character of the double β-barrel is present in most polintons/mavericks sequences [48]—polintons will be also included in the phylogentic-tree of this lineage.

**Figure 3:**
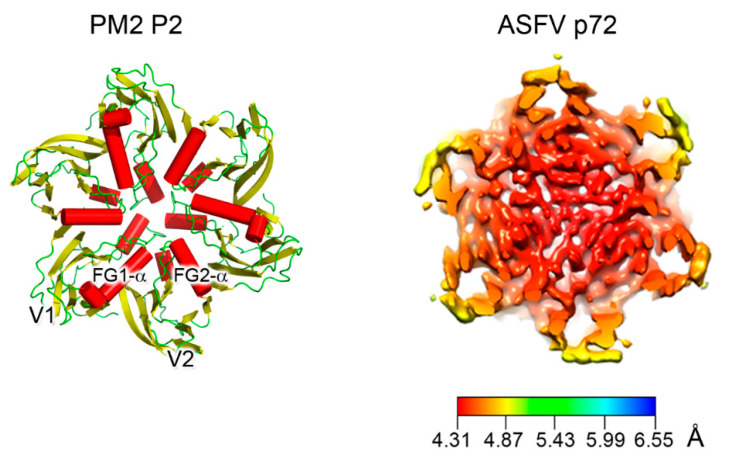
Double β-barrel invariant motifs. Left, view from the top of the pseudo-hexameric capsomer formed by the three copies of PM2 MCP P2 (PDBid 2W0C)—the minimalist of the vertical DJR—with red cylinders representing the α-helices. The estimated angle between the two helices FG1-α and FG2-α is ~72° in line to that found across the corresponding helices in PRD1. Right, cryo-EM map of homotrimers of ASFV MCP p72 viewed along the 3-fold axis showing the pseudo-hexameric footprint generated by the p72 protein adopting the double jelly-roll fold (EMDBid 10325); this is displayed using the local resolution and mapped onto the density (color-coded resolution bar in Å) [52].

The size distribution of member viruses of the above lineage (from ~600 to ~2400 Å in diameter) shows how the pseudo-hexameric morphology of the capsomers is elegantly used to fulfill the requirement for building an icosahedron according to the assembly principles proposed by Caspar and Klug [49,50]. The footprint of these capsomers, which occupy the hexavalent positions on a planar hexagonal lattice, is an invariant trait of this lineage and measures approximately 75 Å in diameter (Figure 3, left) [33]. This dimension ultimately recapitulates the relative packing and angular orientation of the individual β-barrels, V1 and V2, which is reflected in the angular aperture, of helices FG1-α and FG2-α (~69° as estimated for PRD1 P3 in CHIMERA [23,51]) and is mainly conserved across all the PRD1-adenovirus members (Figure 2B and Figure 3; <66.9° > ±7.3° as estimated across 26 MCP structures derived either through X-ray or cryo-EM). The structural invariance of this footprint allows the identification of the double β-barrel fold even at intermediate resolution, as occurred in the case of the African Swine Fever virus (ASFV) p72 MCP whose 8280 copies compose the virion outermost capsid shell (Figure 3, right) [52]. The ASFV p72 double β-barrel and the MCPs of the other large nucleocytoplasmic large DNA virus (NCLDV), PBCV-1 and Faustovirus cluster together [26,33,53,54,55,56] (Figure 2B). This NCLDV clustering indicates that some structural differences in the core consensus DJR exist when compared to the DJR core of the smaller MCPs of PRD1 or PM2. Structural alignment carried out with the HSF software [17] highlights that the two V1 and V2 jelly rolls in NCLDV MCPs are further spaced. Three β-sheets at the base of, and connecting, the two jelly rolls appear to act as a spacer between the two domains. Except PM2 P2, which represents the minimalist double β-barrel core module, all MCPs are crowned by large and structured insertions in loops connecting, for example, the DE and FG β-strands above the two V1 and V2 β-barrels, and have extensions at the N- and C-terminal ends (Figure 1B and Figure 2B). The former protrude toward the outside of the virus while the latter point to the interior of the virion leading to a three module structure (Figure 2B; e.g., adenovirus); each module with specific functionalities. The core double β-barrel is key for assembling the pseudo-hexameric capsomers, the elemental building blocks for generating virions with increasing sizes (Figure 1C). The interior-module allows the rotational registering across capsomers and the anchoring of the capsomers to the underneath structural constituents and, depending on the virus type, to the membrane vesicle, to membrane-associated proteins, or to the genome (Figure 1B, left) [27,28,29,57]. The exterior module, constituted by the turret domains or extended loops above the V1 and V2 towers, is also in some cases, such as adenovirus hexon, faustovirus and ASFV p72 MCPs, involved in trimer/capsomer stabilization [22,33,53]. However, in the case of adenovirus hexon (Figure 1B), it has been shown that these structural elements extending outward from the virion surface contain the so-called “hypervariable regions”, which play a crucial role in eliciting the cell immune response [58]. These sites offer the possibility to be genetically modified or re-purposed with important consequences in vaccine and gene therapy applications in cases of pre-existing immunity or the delivery is hampered for specific adeno-vector serotypes [59,60].

The PRD1-adenovirus lineage is expanding; a new tentative family of viruses called *Autolykiviridae* may also possess a MCP with a DJR fold [61]. Within the current ICTV 15-rank classification, the PRD1-adenovirus structure-based lineage constitutes the new taxon rank of Kingdom with *Bamfordvirae* as taxon name (for the reasons behind this name please see Dennis Bamford’s webpage, Helsinki University), which in turn, falls within the Realm of the *Varidnaviria* [44,45].

## 4. Viruses with Vertical Single β-barrel MCPs: A Clade of the PRD1-Adenovirus Lineage

In 2006, the identification of icosahedral archaeal tailless membrane-containing *Haloarcula hispanica* virus 1 (SH1) encoding two MCPs but forming capsomers with a resemblance to that of PRD1 (e.g., similar pseudo-hexagonal footprint) raised the question of how the virus would assemble [62,63]. SH1 pseudo-hexameric capsomers displayed both two- and three-turret morphologies whilst capsomers of *Thermus* bacteriophage P23-77, at the time the only other virus identified possessing two MCPs, a two-turret morphology. Both viruses share the same triangulation (*T*) number, pseudo *T* = 28. When the X-ray structures of VP16 and VP17 MCPs of P23-77 were solved, they showed that each of the MCPs adopts a vertical β-barrel (or vertical single jelly-roll, vSJR) fold. Later, the characterization at 11 Å by cryo-EM of the archaeal lipid-containing *Haloarcula hispanica* icosahedral virus 2 (HHIV-2) suggested not only MCPs with a vSJR fold but also the presence of additional proteins in between the capsid and the membrane vesicle [64,65]. None of the above studies, however, explained the assembly process of these new viruses for which a novel taxonomic family, *Spherolipoviridae*, was approved by the ICTV in 2015 [39,66].

**Figure 4:**
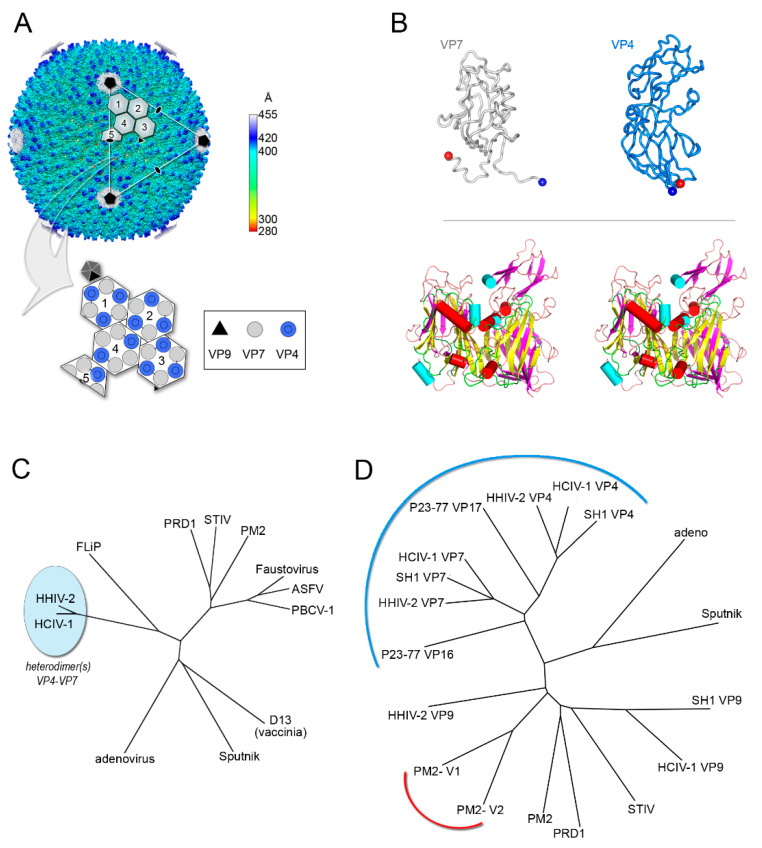
Vertical single β-barrel viruses. (**A**) Overall view of the cryo-EM map of HCIV-1 at 3.74 Å resolution colored by radius as from side bar; the white capsomers numbered from 1 to 3 show a pseudo-hexameric, three-turreted morphology, while capsomer 4 and half-capsomer 5 (sitting on the two-fold axis of symmetry) show a pseudo-hexameric, two-turreted morphology. Capsomers 1–5, together with one copy of the penton protein, compose the icosahedral asymmetric unit (IAU). The white triangle indicates a virus facet and black pentagons, ovals, and triangle mark the 5-fold, 2-fold, and 3-fold axes of icosahedral symmetry, respectively. The large white outlined arrow points at the schematic representation of the IAU with the organization of the individual MCPs depicted as circles (12 copies of turreted VP4 in blue and 15 copies of VP7 in gray) forming the differently numbered pseudo-hexameric capsomers, one copy of the penton protein VP9 as a black triangle, and the remaining four copies of VP9 sitting on the five-fold axis in dark gray. (**B**) Top, cartoon-tube representation of the heterodimers formed by the VP4 and VP7 vertical single jelly rolls; blue and red spheres denote the N-terminus and C-terminus, respectively. Bottom, stereo superimposition of the vertical double β-barrel formed by the VP4–VP7 heterodimer displayed by secondary structural elements (β-strands magenta, α-helices cyan cylinders, loops/turns pink) with the PM2 P2 MCP (β-strands yellow, α-helices red cylinders, loops/turns green). (**C**) Structure-based phylogenetic tree as in Figure 2B but including the chimeric double jelly roll (DJR) generated by the heterodimer VP7-VP4. (**D**) Structure-based phylogenetic tree of vertical single jelly rolls (vSJR) used for the assembly of the pseudo-hexameric capsomers and the penton compared with the individual minimalist V1 and V2 jelly rolls of PM2 P2 MCP; the blue arch line marks the vSJR used for pseudo-capsomer assembly, the red line the individual V1 and V2 jelly rolls of the DJR of PM2 P2 MCP and the remaining the SJR used for penton formation at the 5-fold icosahedral axes (evolutionary distances calculated with the SHP software [16]).

Their assembly mechanism was finally unraveled in 2019 when the cryo-EM structures of the latest discovered membrane-containing archaeal *Haloarcula californiae* icosahedral virus 1 (HCIV-1), HHIV-2 and SH1 were determined at ~3.8 Å resolution (Figure 4A,B) [67,68]. The virions’ structures showed two distinct proteins with an α- and an α/β-fold located beneath the two- and three-tower capsomers, respectively, and homopentameric membrane proteins at the vertices [67]. They orchestrate the positioning relative to the membrane of penton proteins (VP9) and the pre-formed vertical single β-barrel MCP heterodimers (VP4 and VP7), acting as global-positioning-system (GPS) proteins [67]. These heterodimers (at least in HCIV-1, HHIV-2 and SH1) mimic the relative angular orientation of the individual β-barrels in DJR MCPs of the PRD1-adenovirus lineage (Figure 4B,C). However, the characteristics FG1-α and FG2-α helices which are common motifs in all DJR (Figure 1B, Figure 2B and Figure 3, left) are not equivalently oriented in the vSJR MCPs known so far (Figure 4B, bottom). Their different length and orientation, in particular for the vSJR VP7 may reflect the requirement of a fine tuning in the formation of heterodimers and consequently in the rotational registering and packing of the individual jelly-roll forming the pseudo-hexameric capsomers (Figure 4C). Structural superimposition of the vSJR MCPs onto the individual V1 and V2 jelly-roll of the minimalist DJR PM2 P2 and the vertical β-barrel proteins composing the penton of the vertical DJR and SJR viruses groups the vSJR used for the formation of the capsomers apart from those used for the penton, albeit with the PM2 P2 V1 and V2 closer to certain penton proteins (Figure 4D). This clustering supports the idea of a specialization of interactions according to their location within the capsid shell.

Thus, assembly of viruses with two vertical single β-barrel MCPs relies, apart from the protein at the vertices, on homo- and/or hetero-dimerization motifs in the MCPs and on additional molecular guiding proteins in between the capsid protein shell and the outer membrane leaflet. The fusion event of the two consecutive MCP genes leading to the double jelly rolls not only simplify the assembly but more importantly appear to represent so far the sine qua non for the uncoupling of the assembly from the presence of the membrane as seen in adenovirus [67].

It remains to be seen whether one day we will identify a virus with only one vertical single jelly-roll MCP capable of forming pseudo-hexameric capsomers prefiguring the assembly of viruses with two vertical single jelly rolls.

## 5. The Open Question of A Structure-Based Classification of Enveloped Viruses and the Example of Current SARS-CoV-2

**Figure 5:**
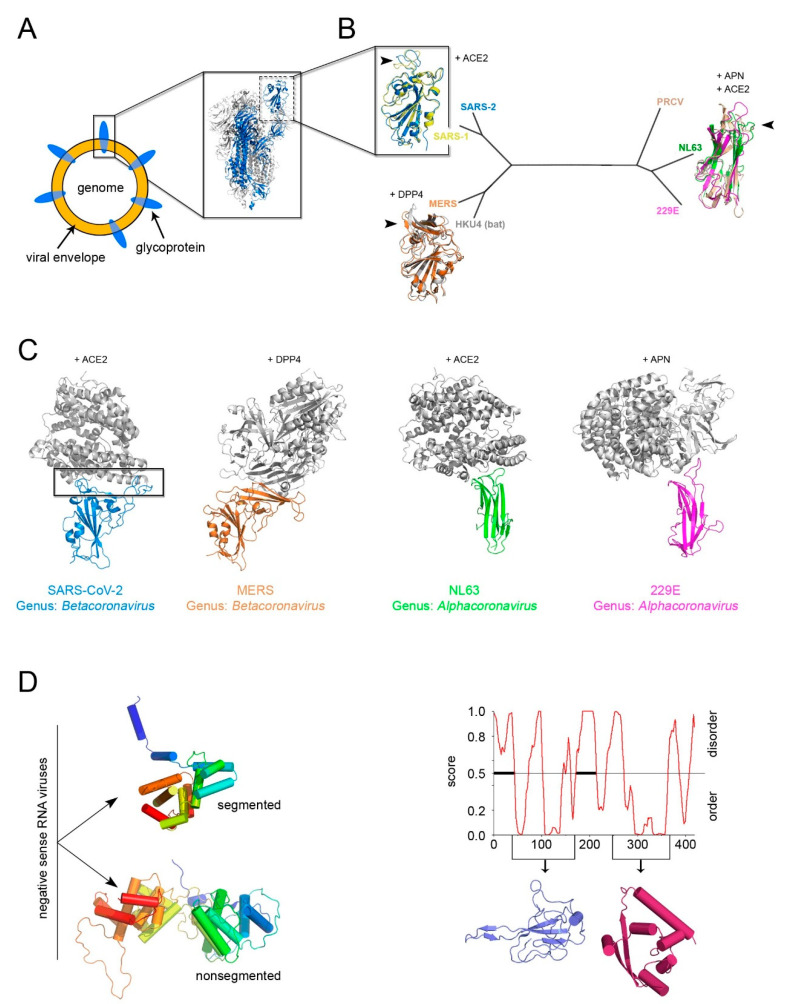
The conundrum of whether a constituent structural element (“self”) for enveloped viruses exists, the example of Coronaviruses. (**A**) Simplified representation of an enveloped virus with the inset depicting the recently determined SARS-CoV-2 S glycoprotein forming the trimeric spike complex (cartoon represented with 2 copies in white and one in marine blue; PDBid 6VXX); the dashed rectangle marks the RBD domain. (**B**) Structured-based phylogenetic-tree of representative coronavirus RBDs determined in complex with corresponding cellular receptors (SARS-2, PDBid 6LZG; SARS-1, PDBid 3D0I; MERS, PDBid 4KR0; HKU4, PDBid 4QZV; PRCV, PDBid 4F5C; NL63, PDBid 3KBH; 229E, PDBid 6ATK) showing the clustering of the RBDs in two distant folds; in cartoon representation, the superimposition of the corresponding RBDs color-coded according to the belonging virion with the receptors to which they bind shown. The black arrowed-head marks the hot-spot regions for evolution malleability. (**C**) To-scale and equivalently oriented comparisons of the host-spots viral binding regions (marked by a black rectangle in the SARS-CoV-2) with their respective cellular receptors in cartoon and color-coded as in B. Based on the phylogeny of viral RdRp, the members of the subfamily *Orthocoronavirinae* (within the *Coronaviridae* family) have been clustered in four genera: *Alphacoronavirus*, *Betacoronavirus*, *Gammacoronavirus*, and *Deltacoronovirus*. (**D**) Left, representative different nucleocapsid protein folds from Rift Valley fever virus (PDBid 3OV9) (top) and Rabies virus (PDBid 2GTT) (bottom), respectively (for details see [38]), for the non-segmented and segmented negative sense RNA represented by secondary structure and color-coded in rainbow from blue to red from N- to C-terminal ends. Right, PONDr disordered analysis (http://www.pondr.com/) of the SARS-CoV-2 nucleoprotein with below depicted the domains that have been determined by X-ray crystallography (PDBids 6VYO and 6YUN) but which do not bear any resemblance with the structures depicted on the left panel [73].

The identification of a structural “self” in enveloped viruses remains a challenge [5,38]. While glycoproteins decorating the viral envelope are usually categorized in three structural classes of fusion proteins (I–III) [69], their fold is a weak determinant for inferring distant viral relationships (Figure 5A). Firstly, it has been shown that class II viral glycoproteins have counterparts in cellular proteins, demonstrating the possibility of horizontal gene exchange [70], and secondly, viral glycoproteins are under environmental selection pressure for the cellular receptors and thus inclined to higher sequence and/or structural variability. This is noticeable when comparing the available structures of the receptor-binding domain (RBD) within the spike glycoprotein S (a class I viral fusogen) across the members of the *Coronaviridae* family, as shown early this year [71,72].

However, with the emerging of SARS-CoV-2, a further manifestation of evolution at work, the power of sequence and structural analysis has revealed in record time the determinants for the recognition of the RBD for the human angiotensin-converting enzyme 2 (ACE2) [74,75]. The known RBDs adopt either a twisted five-stranded antiparallel β-sheet with short connecting helices and loops or a β-sandwich (Figure 5B). Superimposition of the available RBD-receptor complexes highlights the loops connecting the β-strands as the regions more susceptible to retain mutations (so-called receptor-binding-motifs) that render the RBD the master key for operating different cellular “locks” (e.g., ACE2, aminopeptidase N (APN), dipeptidyl peptidase 4 (DDP4) but also 9-*O*-acetylated neuraminic acid, carcinoembryonic antigen-related adhesion molecule 1, heparin sulfate, and α-2,3-linked sialic acid [76]) (Figure 5B,C).

Thus, the flexible loops not strictly possessing a structural role in the (RBD) protein fold are more prone to accommodate amino acid changes and possible elaborations (Figure 5B,C) as similarly observed in the conformational diversity of the connecting loops at the top of the vDJR/vSJR of the PRD1-adenovirus lineage (Figure 2B).

In the search of “self” candidates, the fold of the nucleocapsid protein has also been considered. In the case of negative-sense RNA viruses two distinct α-helical folds for this protein could cluster apart viruses with segmented genome from those with non-segmented genome (Figure 5C, left) [38]. For other enveloped viruses, a classification based on the nucleocapsid protein structure remains arduous. Therefore, whether the nucleocapsid is a reliable self for inferring common ancestry remains to be seen.

As for positive-sense ssRNA SARS-CoV-2 virus, the RNA binding-domain and the C-terminal domain of the nucleocapsid have been structurally determined but neither of them displays a topological resemblance to those of other viral families (Figure 5C, right) [73]. Its sequence, however, is highly conserved among human coronaviruses (25% < identity < 91%) making the nucleocapsid a cross-reactive antigen in immunological tests of patient sera as opposed to the specificity shown by the SARS-CoV-2 RBD antigen (unpublished).

Finally, all RNA viruses (enveloped and not) and reverse-transcribing viruses encode respectively an RNA-dependent RNA polymerase (RdRp) and a reverse-transcriptase, which allows for these genes to be used as markers in phylogenomic analyses [40]. Both above enzymes as well as the gene encoding for DJR MCPs have been suggested as “hallmark genes” for the virus classification currently adopted by the ICTV [45].

## 6. Conclusions

The journey of discovering evolutionary relationships through a structure-based approach across different viruses has not stopped since the initial observation that some animal and plant RNA viruses MCPs—with limited sequence identity—share a similar fold [20]. In fact, this journey is experiencing a rapid acceleration with the increased capabilities in genome sequencing, in structure determination and in more sensitive phylogenetic methods.

Deciphering sequence and structural homologies does not only emerge from the need of the human mind to categorize the complexity that surrounds us as a means to understand it, but also from its power in impacting on the fight against viral diseases.

## References

[1] Gago S., Elena S.F., Flores R., Sanjuan R. (2009). Extremely high mutation rate of a hammerhead viroid. Science.

[2] Koonin E.V., Galperin M.Y. (2003). Evolutionary concept in genetics and genomics. Sequence Evolution Function: Computational Approaches in Comparative Genomics.

[3] Kimura K., Hunley S.B., Namy L.L. (2018). Children’s use of comparison and function in novel object categorization. J. Exp. Child Psychol..

[4] Ravantti J., Abrescia N.G., Bamford D., Zuckerman M. (2021). Classification of the viral world based on atomic level structures. Encyclopedia of Virology.

[5] Abrescia N., Bamford D., Zuckerman M. (2021). Evolution steered by structure. Encyclopedia of Virology.

[6] Kuhlbrandt W. (2014). Biochemistry. The resolution revolution. Science.

[7] Stuart D.I., Subramaniam S., Abrescia N.G. (2016). The democratization of cryo-EM. Nat. Methods.

[8] Perutz M.F., Rossmann M.G., Cullis A.F., Muirhead H., Will G., North A.C. (1960). Structure of haemoglobin: A three-dimensional Fourier synthesis at 5.5-Å. resolution, obtained by X-ray analysis. Nature.

[9] Perutz M.F., Muirhead H., Cox J.M., Goaman L.C. (1968). Three-dimensional Fourier synthesis of horse oxyhaemoglobin at 2.8 Å resolution: The atomic model. Nature.

[10] Huber R., Epp O., Steigemann W., Formanek H. (1971). The atomic structure of erythrocruorin in the light of the chemical sequence and its comparison with myoglobin. Eur. J. Biochem..

[11] Rao S.T., Rossmann M.G. (1973). Comparison of super-secondary structures in proteins. J. Mol. Biol..

[12] Rossmann M.G., Argos P. (1976). Exploring structural homology of proteins. J. Mol. Biol..

[13] Sali A., Blundell T.L. (1990). Definition of general topological equivalence in protein structures. A procedure involving comparison of properties and relationships through simulated annealing and dynamic programming. J. Mol. Biol..

[14] Krissinel E., Henrick K. (2004). Secondary-structure matching (SSM), a new tool for fast protein structure alignment in three dimensions. Acta Crystallogr. Sect. D Biol. Crystallogr..

[15] Holm L., Sander C. (1993). Protein structure comparison by alignment of distance matrices. J. Mol. Biol..

[16] Stuart D.I., Levine M., Muirhead H., Stammers D.K. (1979). Crystal structure of cat muscle pyruvate kinase at a resolution of 2.6 Å. J. Mol. Biol..

[17] Ravantti J., Bamford D., Stuart D.I. (2013). Automatic comparison and classification of protein structures. J. Struct. Biol..

[18] Harrison S.C., Olson A.J., Schutt C.E., Winkler F.K., Bricogne G. (1978). Tomato bushy stunt virus at 2.9 Å resolution. Nature.

[19] Abad-Zapatero C., Abdel-Meguid S.S., Johnson J.E., Leslie A.G., Rayment I., Rossmann M.G., Suck D., Tsukihara T. (1980). Structure of southern bean mosaic virus at 2.8 Å resolution. Nature.

[20] Rossmann M.G., Arnold E., Erickson J.W., Frankenberger E.A., Griffith J.P., Hecht H.J., Johnson J.E., Kamer G., Luo M., Mosser A.G. (1985). Structure of a human common cold virus and functional relationship to other picornaviruses. Nature.

[21] Hogle J.M., Chow M., Filman D.J. (1985). Three-dimensional structure of poliovirus at 2.9 Å resolution. Science.

[22] Burnett R.M., Athappilly F.K., Cai Z., Furcinitti P.S., Korn A.P., Murali R., van Oostrum J. (1990). Structure of the Adenovirus Virion. Use of X-ray Crystallography in the Design of Antiviral Agents.

[23] Abrescia N.G., Cockburn J.J.B., Grimes J.M., Sutton G.C., Diprose J.M., Butcher S.J., Fuller S.D., San Martín C., Burnett R.M., Stuart D.I. (2004). Insights into assembly from structural analysis of bacteriophage PRD1. Nature.

[24] Cockburn J.J., Abrescia N.G., Grimes J.M., Sutton G.C., Diprose J.M., Benevides J.M., Thomas G.J., Bamford J.K.H., Bamford D.H., Stuart D.I. (2004). Membrane structure and interactions with protein and DNA in bacteriophage PRD1. Nature.

[25] Chapman M.S., Liljas L. (2003). Structural folds of viral proteins. Adv. Protein Chem..

[26] Benson S.D., Bamford J.K., Bamford D.H., Burnett R.M. (1999). Viral evolution revealed by bacteriophage PRD1 and human adenovirus coat protein structures. Cell.

[27] Bamford D.H., Burnett R.M., Stuart D.I. (2002). Evolution of viral structure. Theor. Popul. Biol..

[28] Nandhagopal N., Simpson A.A., Gurnon J.R., Yan X., Baker T.S., Graves M.V., Van Etten J.L., Rossmann M.G. (2002). The structure and evolution of the major capsid protein of a large, lipid-containing DNA virus. Proc. Natl. Acad. Sci. USA.

[29] Abrescia N.G., Grimes J.M., Kivelä H.M., Assenberg R., Sutton G.C., Butcher S.J., Bamford J.K.H., Bamford D.H., Stuart D.I. (2008). Insights into virus evolution and membrane biogenesis from the structure of the marine lipid-containing bacteriophage PM2. Mol. Cell.

[30] Fokine A., Leiman P.G., Shneider M.M., Ahvazi B., Boeshans K.M., Steven A.C., Black L.W., Mesyanzhinov V.V., Rossmann M.G. (2005). Structural and functional similarities between the capsid proteins of bacteriophages T4 and HK97 point to a common ancestry. Proc. Natl. Acad. Sci. USA.

[31] Pietila M.K., Laurinmäki P., Russell D.A., Ko C.-C., Jacobs-Sera D., Hendrix R.W., Bamford D.H., Butcher S.J. (2013). Structure of the archaeal head-tailed virus HSTV-1 completes the HK97 fold story. Proc. Natl. Acad. Sci. USA.

[32] Bahar M.W., Graham S.C., Stuart D.I., Grimes J.M. (2011). Insights into the evolution of a complex virus from the crystal structure of vaccinia virus d13. Structure.

[33] Klose T., Reteno D.G., Benamar S., Hollerbach A., Colson P., La Scola B., Rossmann M.G. (2016). Structure of faustovirus, a large dsDNA virus. Proc. Natl. Acad. Sci. USA.

[34] Khayat R., Tang L., Larson E.T., Lawrence C.M., Young M., Johnson J.E. (2005). Structure of an archaeal virus capsid protein reveals a common ancestry to eukaryotic and bacterial viruses. Proc. Natl. Acad. Sci. USA.

[35] Hyun J.K., Accurso C., Hijnen M., Schult P., Pettikiriarachchi A., Mitra A.K., Coulibaly F. (2011). Membrane remodeling by the double-barrel scaffolding protein of poxvirus. PLoS Pathog..

[36] Duda R.L., Teschke C.M. (2019). The amazingHK97 fold: Versatile results of modest differences. Curr. Opin. Virol..

[37] Abrescia G., Twarock P.G.S.R. (2010). Fry, Ravantti, Bamford, Stuart, What does it take to make a virus: The concept of the viral “self”. Emerging Topics in Physical Virology.

[38] Abrescia N.G., Bamford D.H., Grimes J.M., Stuart D.I. (2012). Structure Unifies the Viral Universe. Annu. Rev. Biochem..

[39] Lefkowitz E.J., Dempsey D.M., Hendrickson R.C., Orton R.J., Siddell S.G., Smith D.B. (2018). Virus taxonomy: The database of the International Committee on Taxonomy of Viruses (ICTV). Nucleic Acids Res..

[40] Wolf Y.I., Kazlauskas D., Iranzo J., Lucía-Sanz A., Kuhn J.H., Krupovic M., Dolja V.V., Koonin E.V. (2018). Origins and Evolution of the Global RNA Virome. mBio.

[41] Koonin E.V., Senkevich T.G., Dolja V.V. (2006). The ancient Virus World and evolution of cells. Biol. Direct..

[42] Krupovic M., Dolja V.V., Koonin E.V. (2019). Origin of viruses: Primordial replicators recruiting capsids from hosts. Nat. Rev. Microbiol..

[43] Krupovic M., Koonin E.V. (2017). Multiple origins of viral capsid proteins from cellular ancestors. Proc. Natl. Acad. Sci. USA.

[44] International Committee on Taxonomy of Viruses Executive Committee (2020). The new scope of virus taxonomy: Partitioning the virosphere into 15 hierarchical ranks. Nat. Microbiol..

[45] Koonin E.V., Dolja V.V., Krupovic M., Varsani A., Wolf Y.I., Yutin N., Zerbini F.M., Kuhn J.H. (2020). Global Organization and Proposed Megataxonomy of the Virus World. Microbiol. Mol. Biol. Rev..

[46] Laanto E., Mäntynen S., De Colibus L., Marjakangas J., Gillum A., Stuart D.I., Ravantti J.J., Huiskonen J.T., Sundberg L.R. (2017). Virus found in a boreal lake links ssDNA and dsDNA viruses. Proc. Natl. Acad. Sci. USA.

[47] Zhang X., Sun S., Xiang Y., Wong J., Klose T., Raoult D., Rossmann M.G. (2012). Structure of Sputnik, a virophage, at 3.5-A resolution. Proc. Natl. Acad. Sci. USA.

[48] Krupovic M., Koonin E.V. (2015). Polintons: A hotbed of eukaryotic virus, transposon and plasmid evolution. Nat. Rev. Microbiol..

[49] Caspar D.L., Klug A. (1962). Physical principles in the construction of regular viruses. Cold Spring Harb. Symp. Quant. Biol..

[50] Simpson A.A., Nandhagopal N., Van Etten J.L., Rossmann M.G. (2003). Structural analyses of Phycodnaviridae and Iridoviridae. Acta Crystallogr. D Biol. Crystallogr..

[51] Pettersen E.F., Goddard T.D., Huang C.C., Couch G.S., Greenblatt D.M., Meng E.C., Ferrin T.E. (2004). UCSF Chimera—A visualization system for exploratory research and analysis. J. Comput. Chem..

[52] Andres G., Charro D., Matamoros T., Dillard R.S., Abrescia N.G. (2020). The cryo-EM structure of African swine fever virus unravels a unique architecture comprising two icosahedral protein capsids and two lipoprotein membranes. J. Biol. Chem..

[53] Liu Q., Ma B., Qian N., Zhang F., Tan X., Lei J., Xiang Y. (2019). Structure of the African swine fever virus major capsid protein p72. Cell Res..

[54] Liu S., Luo Y., Wang Y., Li S., Zhao Z., Bi Y., Sun J., Peng R., Song H., Zhu D. (2019). Cryo-EM Structure of the African Swine Fever Virus. Cell Host Microbe.

[55] Wang N., Zhao D., Wang J., Zhang Y., Wang M., Gao Y., Li F., Wang J., Bu Z., Rao Z. (2019). Architecture of African swine fever virus and implications for viral assembly. Science.

[56] Fang Q., Zhu D., Agarkova I., Adhikari J., Klose T., Liu Y., Chen Z., Sun Y., Gross M.L., Van Etten J.L. (2019). Near-atomic structure of a giant virus. Nat. Commun..

[57] Born D., Reuter L., Mersdorf U., Mueller M., Fischer M.G., Meinhart A., Reinstein J. (2018). Capsid protein structure, self-assembly, and processing reveal morphogenesis of the marine virophage mavirus. Proc. Natl. Acad. Sci. USA.

[58] Rux J.J., Burnett R.M. (2000). Type-specific epitope locations revealed by X-ray crystallographic study of adenovirus type 5 hexon. Mol. Ther..

[59] Schmid M., Ernst P., Honegger A., Suomalainen M., Zimmermann M., Braun L., Stauffer S., Thom C., Dreier B., Eibauer M. (2018). Adenoviral vector with shield and adapter increases tumor specificity and escapes liver and immune control. Nat. Commun..

[60] Prill J.M., Espenlaub S., Samen U., Engler T., Schmidt E., Vetrini F., Rosewell A., Grove N., Palmer D., Ng P. (2011). Modifications of adenovirus hexon allow for either hepatocyte detargeting or targeting with potential evasion from Kupffer cells. Mol. Ther..

[61] Kauffman K.M., Hussain F.A., Yang J., Arevalo P., Brown J.M., Chang W.K., VanInsberghe D., Elsherbini J., Sharma R.S., Cutler M.B. (2018). A major lineage of non-tailed dsDNA viruses as unrecognized killers of marine bacteria. Nature.

[62] Porter K., Kukkaro P., Bamford J.K., Bath C., Kivelä H.M., Dyall-Smith M.L., Bamford D.H. (2005). SH1: A novel, spherical halovirus isolated from an Australian hypersaline lake. Virology.

[63] Kivela H.M., Roine E., Kukkaro P., Laurinavicius S., Somerharju P., Bamford D.H. (2006). Quantitative dissociation of archaeal virus SH1 reveals distinct capsid proteins and a lipid core. Virology.

[64] Rissanen I., Pawlowski A., Harlos K., Grimes J.M., Stuart D.I., Bamford J.K.H. (2012). Crystallization and preliminary crystallographic analysis of the major capsid proteins VP16 and VP17 of bacteriophage P23-77. Acta Crystallogr..

[65] Gil-Carton D., Jaakkola S.T., Charro D., Peralta B., Castaño-Díez D., Oksanen H.M., Bamford D.H., Abrescia N.G. (2015). Insight into the Assembly of Viruses with Vertical Single beta-barrel Major Capsid Proteins. Structure.

[66] Pawlowski A., Rissanen I., Bamford J.K., Krupovic M., Jalasvuori M. (2014). Gammasphaerolipovirus, a newly proposed bacteriophage genus, unifies viruses of halophilic archaea and thermophilic bacteria within the novel family Sphaerolipoviridae. Arch. Virol..

[67] Santos-Pérez I., Charro D., Gil-Carton D., Azkargorta M., Elortza F., Bamford D.H., Oksanen H.M., Abrescia N.G. (2019). Structural basis for assembly of vertical single beta-barrel viruses. Nat. Commun..

[68] De Colibus L., Roine E., Walter T.S., Ilca S.L., Wang X., Wang N., Roseman A.M., Bamford D.H., Huiskonen J.T., Stuart D.I. (2019). Assembly of complex viruses exemplified by a halophilic euryarchaeal virus. Nat. Commun..

[69] Harrison S.C. (2015). Viral membrane fusion. Virology.

[70] Pérez-Vargas J., Krey T., Valansi C., Avinoam O., Haouz A., Jamin M., Raveh-Barak H., Podbilewicz B., Rey F.A. (2014). Structural basis of eukaryotic cell-cell fusion. Cell.

[71] Bosch B.J., van der Zee R., de Haan C.A., Rottier P.J. (2003). The coronavirus spike protein is a class I virus fusion protein: Structural and functional characterization of the fusion core complex. J. Virol..

[72] Ng W.M., Stelfox A.J., Bowden T.A. (2020). Unraveling virus relationships by structure-based phylogenetic classification. Virus Evol..

[73] Ye Q., West A.M.V., Silletti S., Corbett K.D. (2020). Architecture and self-assembly of the SARS-CoV-2 nucleocapsid protein. Protein Sci..

[74] Shang J., Ye G., Shi K., Wan Y., Luo C., Aihara H., Geng Q., Auerbach A., Li F. (2020). Structural basis of receptor recognition by SARS-CoV-2. Nature.

[75] Lan J., Ge J., Yu J., Shan S., Zhou H., Fan S., Zhang Q., Shi X., Wang Q., Zhang L. (2020). Structure of the SARS-CoV-2 spike receptor-binding domain bound to the ACE2 receptor. Nature.

[76] Belouzard S., Millet J.K., Licitra B.N., Whittaker G.R. (2012). Mechanisms of coronavirus cell entry mediated by the viral spike protein. Viruses.

